# Simulating the healthcare workforce impact and capacity for pancreatic cancer care in Victoria: a model-based analysis

**DOI:** 10.1186/s12913-024-10722-9

**Published:** 2024-02-23

**Authors:** Lan Gao, Anna Ugalde, Patricia M Livingston, Victoria White, Jennifer J Watts, Hannah Jongebloed, Nikki McCaffrey, David Menzies, Suzanne Robinson

**Affiliations:** 1 Deakin Health Economics, Institute of Health Transformation, Faculty of Health, Deakin University, 1 Gheringhap St, 3220 Geelong, Australia; 2 School of Nursing & Midwifery, Institute of Health Transformation, Faculty of Health, Deakin University, Melbourne, Australia; 3 PanSupport Pancare Foundation, Melbourne, Australia

**Keywords:** Pancreatic cancer, Screening tool, Workforce planning, Decision-making, Modelling

## Abstract

**Background:**

The incidence of pancreatic cancer is rising. With improvements in knowledge for screening and early detection, earlier detection of pancreatic cancer will continue to be more common. To support workforce planning, our aim is to perform a model-based analysis that simulates the potential impact on the healthcare workforce, assuming an earlier diagnosis of pancreatic cancer.

**Methods:**

We developed a simulation model to estimate the demand (i.e. new cases of pancreatic cancer) and supply (i.e. the healthcare workforce including general surgeons, medical oncologists, radiation oncologists, pain medicine physicians, and palliative care physicians) between 2023 and 2027 in Victoria, Australia. The model compares the current scenario to one in which pancreatic cancer is diagnosed at an earlier stage. The incidence of pancreatic cancer in Victoria, five-year survival rates, and Victoria’s population size were obtained from Victorian Cancer Registry, Cancer Council NSW, and Australian Bureau of Statistics respectively. The healthcare workforce data were sourced from the Australian Government Department of Health and Aged Care’s Health Workforce Data. The model was constructed at the remoteness level. We analysed the new cases and the number of healthcare workforce by profession together to assess the impact on the healthcare workforce.

**Results:**

In the status quo, over the next five years, there will be 198 to 220 stages I-II, 297 to 330 stage III, and 495 to 550 stage IV pancreatic cancer cases diagnosed annually, respectively. Assuming 20–70% of the shift towards pancreatic cancer’s earlier diagnosis (shifting from stage IV to stages I-II pancreatic cancer within one year), the stages I-II cases could increase to 351 to 390 or 598 to 665 per year. The shift to early diagnosis led to substantial survival gains, translating into an additional 284 or 795 out of 5246 patients with pancreatic cancer remaining alive up to year 5 post-diagnosis. Workforce supply decreases significantly by the remoteness levels, and remote areas face a shortage of key medical professionals registered in delivering pancreatic cancer care, suggesting travel necessities by patients or clinicians.

**Conclusion:**

Improving the early detection and diagnosis of pancreatic cancer is expected to bring significant survival benefits, although there are workforce distribution imbalances in Victoria that may affect the ability to achieve the anticipated survival gain.

**Supplementary Information:**

The online version contains supplementary material available at 10.1186/s12913-024-10722-9.

## Background

Internationally, the increased incidence of pancreatic cancer suggests it will soon be a major cause of cancer-related deaths in several parts of the world, including central Europe, North America, Western Europe, and Southern Latin America [1]. Pancreatic cancer is a significant health issue in Australia, representing the third most deadly form of cancer despite being the eighth most prevalent type with over 4,000 people diagnosed with pancreatic cancer in 2021 (a quarter of whom are from Victoria) and over 3,000 dying from this cancer [2]. Most cases of pancreatic cancer are diagnosed at an advanced stage. For instance, in the United States, approximately 50% of pancreatic cancers diagnosed from 2009 to 2018 were at an advanced stage, 29% were at a regional stage, and only 12% were at a localised stage [3]. The five-year relative survival rates vary depending on the stage of a pancreatic cancer diagnosis. Those diagnosed with localised tumours have a notably higher five-year relative survival rate (41.6%) than those diagnosed with regional (14.4%) or distant (3.0%) spread of the disease [3, 4]. Despite a marginal increase in the five-year relative survival rate of pancreatic cancer in Australia over the last three decades, from 3.4% (1988–1992) to 11.5% (2013–2017), it remains significantly low compared to the survival rate of 69.7% for all cancers combined [2].

Although early detection of pancreatic cancer can offer numerous advantages [5, 6], population-based screening is not currently recommended because the potential risks associated with screening in asymptomatic adults outweigh the potential benefits, including no survival gains [7, 8–10]. Clinical risk prediction models have been developed for application in different high-risk populations, such as those with a family history of pancreatic cancer, germline mutations, pancreas cystic lesions, new-onset diabetes, and post-pancreatitis diabetes [10–15]. Similar to online cardiovascular disease risk prediction tools, clinical risk factors such as increasing age, male sex, ethnic background, smoking history, alcohol consumption, body mass index, diabetes, chronic pancreatitis, and family history of pancreatic cancer could be employed to identify those at increased risk for pancreatic cancer to initiate further investigation, without relying on imaging modalities [16–25]. Many of these risk prediction tools show promising results, characterised by moderate to strong discrimination performance for pancreatic cancer, indicating their potential clinical usefulness in a targeted screening setting [23].

Australia’s National Pancreatic Cancer Roadmap was developed in 2022, which includes a key objective focused on the early detection of pancreatic cancer through the implementation of pancreatic cancer-specific risk assessment tools in the short term plan [26]. Evidence supports that screen-detected pancreatic cancer in high-risk population are more likely to be resectable (60–90%) than sporadic cases in the general population (15%), representing a potential shift of 45–75% towards the early stages [27–31]. A pancreatic cancer screening tool plays a vital role in identifying people at risk, there is also opportunity to assess the potential workforce impact. Workforce supply and demand modelling is a valuable tool for informing human resource planning, policy formulation, and decision-making in healthcare. An important advantage of utilising such models is their ability to identify any disparities that may exist between the current workforce and the projected future requirements, thereby facilitating the prediction of training and recruitment needs and guiding workforce planning efforts [32]. In healthcare, workforce modelling is centred around the health professionals with specialist skills needed to manage patients diagnosed at different stages of disease (i.e., surgeons, radiation oncologists, pain management specialists, etc.). This modelling is complicated by potential increases in disease incidence. Therefore, appropriate healthcare workforce planning can result in significant improvements, such as prolonging patients’ lives, decreasing morbidity rates, narrowing health disparities, and more efficiently allocating public funds [33].

Given the pivotal role that effective health workforce planning plays in an efficient healthcare system, this paper aimed to assess new pancreatic cancer cases in relation to health workforce supply by remoteness level, assuming a risk-based screening, to demonstrate the potential impact of a risk tool. With screening programs currently being trialled, this is a timely response to a prossible targeted surveillance program that may be be introduced in Australia in short to medium term. Particularly, we intended to:

1. Identify the predicted workforce supply in the key professions involved in pancreatic cancer treatment and management.
2. Outline the predicted new cases of pancreatic cancers or projected demand.
3. Estimate the additional number of patients who survived up to 5-year in the proposed scenario.

## Methods

### Setting

This modelling was applied to statistics from Victoria, Australia, a state with a population of 6.747 million (second most populous). Approximately 77% of Victorians live in Greater Melbourne and 23% in rural and regional Victoria [34].

### Workforce model

A workforce model was developed to estimate the demand (i.e. predicted new cases of pancreatic cancer) and supply (i.e. the key healthcare workforce involved in treating and managing pancreatic cancer) for the next five years in Victoria (2023–2027), comparing the current scenario to one in which pancreatic cancer is diagnosed at an earlier stage due to introduction of a potential clinical risk assessment tool for targeted screening. The model was set up by remoteness levels in Victoria to provide a geographic picture of the demand and supply in the pancreatic cancer landscape.

### The population size of victoria

Population size in Victoria by remoteness levels, including the trend in changes, was informed by the Australian Bureau of Statistics (ABS) [34]. The trend in population growth has accounted for all-cause mortality and immigration.

### Remoteness levels

Victoria is a geographically diverse state, and to comprehensively assess the relationship between demand and workforce supply, we have employed the Modified Monash Model (MM, 2019 version) to delineate various remoteness levels. This model integrates remoteness population size, and access to health services, resulting in a seven-point scale (MM1 to MM7). MM1 signifies metropolitan areas, while MM7 represents very remote areas with minimum population size. Additional analysis was undertaken by the Local Government Area (covering legally designated parts of a State or Territory for which incorporated local governing bodies have the responsibility, similar to counties in the US. Victoria consists of 80 Local Government Areas).

### Epidemiology of pancreatic cancer

The incidence of pancreatic cancer and five-year survival rates in Victoria over the past 12 years (2010–2021) was sourced from Victorian Cancer Registry [35]. Distribution of diagnostic stage at in current practice was sourced from Cancer Council New South Wales, Australia [36] while five-year survival rate by stages were informed from US data due to the absence of Australian statistics [37]. In the proposed scenario, assumptions were made to shift the distribution of stages at pancreatic cancer diagnosis to model for the potential flow-on impacts of early diagnosis. To be conservative, it was assumed that the incidence of pancreatic cancer remained the same in a given year, but the stage was shifted for the cases within that year [38]. In addition, we tested in the best scenario that early detection could identify pancreatic cancer cases by five years earlier [39].

Incidence of pancreatic cancer, stages at diagnosis, and 5-year survival rate by stages are shown in Supplementary Tables 1 to 3.

### Workforce supply

The health workforce data by profession type was sourced from the Department of Health and Aged Care Health Workforce Data tool [40]. Health workforce data captures the primary registration location of health professionals. While many health professionals practice in multiple locations, these other locations are not noted in the data. Treatment recommendations related to pancreatic cancer in Australia and internationally suggest [41, 42], management of pancreatic cancer is conducted through a multidisciplinary approach, which involves the collaboration of medical oncologists, surgical oncologists, radiation oncologists, gastroenterologists, and other specialists as needed.

In Australia, surgery is considered the primary treatment option for localised pancreatic cancer. The Whipple procedure, or pancreaticoduodenectomy, is a surgical intervention that involves the removal of the pancreatic head, the first segment of the small intestine (duodenum), the gallbladder, and sometimes a portion of the stomach. It is the most commonly performed surgical technique and is considered the standard surgical treatment for localised pancreatic cancer that has not metastasised beyond the pancreas. However, eligibility for surgery may be restricted by factors such as tumour location or patient health status.

Chemotherapy is the primary treatment modality for patients with locally advanced or metastatic pancreatic cancer in Australia [42], For patients with localised advanced pancreatic cancer, radiation therapy alone or in combination with chemotherapy may be a potential treatment option. The present modelling does not examine the workforce impact on pancreatic cancer care in detail (e.g. general practitioners, allied health practitioners, etc.); instead, we focus on the critical medical professions involved in the acute cancer care.

The workforce supply data by profession are listed in Table 1.

**Table 1:** Workforce Supply data for the key health professionals involved in pancreatic cancer treatment^*^

Year	Endocrinology	Gastroenterology and Hepatology	Palliative medicine	Pain medicine	General surgery	Radiation oncology	Medical oncology
*2023*	1862	2274	601	310	4135	914	2104
*2024*	1986	2379	646	336	4248	946	2241
*2025*	2091	2488	695	364	4364	979	2388
*2026*	2202	2603	747	395	4483	1013	2544
*2027*	2318	2723	803	429	4605	1048	2711

*The number of health professionals according to the primary specialty

### Proposed scenario

Targeted surveillance programs have been proposed as a strategy to help identify the disease in high-risk individuals at an early stage (i.e. risk-based screening for pancreatic cancer in the primary care setting, followed by referring high-risk patients on for further investigation as per the diagnostic guidelines) [43]. Subsequently, we have analysed the impact on the healthcare workforce if a higher proportion of patients were diagnosed at an earlier stage of pancreatic cancer. Specifically, we tested three scenarios where 20%, 50%, and 70% of patients were diagnosed in the early stage (Stages I-II) of pancreatic cancer, informed by existing evidence [27–31]. The stage shift by the proposed and current scenarios are shown in Supplementary Table 2.

All the analyses were performed using Microsoft Excel. Tableau (Seattle, WA: Tableau Software) was adopted to plot the demand and supply in Victoria by Local Government Areas.

## Results

### Changes in demand

Over the next five years in Victoria, continuing current diagnostic strategies there will be 198 to 220 stages I-II, and 495 to 550 stage IV pancreatic cancer cases diagnosed annually. Assuming a 70% shift towards pancreatic cancer earlier diagnosis (a strategy that shifts diagnosis from stage III/IV to stages I-II pancreatic cancer in one year), the number of stage I-II cases could increase to between 598 and 665 cases annually, while the number of stage IV cases diagnosed each year reduces to between 149 and 165 cases (Table 2).

**Table 2:** Results of predicted pancreatic cancer cases in Victoria, Australia by scenarios

New cases_status quo				
	Stages I-II	Stage III	Stage IV	Total
*2023*	198	297	495	991
*2024*	206	308	514	1028
*2025*	210	315	526	1051
*2026*	215	323	538	1075
*2027*	220	330	550	1100
**Proposed scenario (20% reduction in current stage IV)**				
	**Stages I-II**	**Stage III**	**Stage IV**	**Total**
*2023*	351	244	396	991
*2024*	364	253	411	1028
*2025*	372	259	420	1051
*2026*	381	265	430	1075
*2027*	390	271	440	1100
**Proposed scenario (50% reduction in current stage IV)**				
	**Stages I-II**	**Stage III**	**Stage IV**	**Total**
*2023*	499	244	248	991
*2024*	518	253	257	1028
*2025*	530	259	263	1051
*2026*	542	265	269	1075
*2027*	555	271	275	1100
**Proposed scenario (70% reduction in current stage IV)** ^*^				
	**Stages I-II**	**Stage III**	**Stage IV**	**Total**
*2023*	598	244	149	991
*2024*	621	253	154	1028
*2025*	635	259	158	1051
*2026*	649	265	161	1075
*2027*	665	271	165	1100
**Proposed scenario (early diagnosis by five years)**				
2023	2119	244	149	2512

*base case

For example, in 2023, under the early diagnosis model with a 70% reduction in stage IV disease, the number of additional cases of stages I-II cases diagnoses ranged from 29 in the medium/small rural towns and beyond, to 324 in the metropolitan area (Table 4). This translated into a total additional 400 stage I-II pancreatic cancer cases, assuming one year of earlier diagnosis, or over 5 years 2119 additional cases in Victoria in the base case (Table 2).

### Changes in survival outcomes

**Table 3:** Results of survival outcomes by scenarios

	Current scenario	Proposed scenario (70% reduction in current stage IV)		Proposed scenario (50% reduction in current stage IV)		Proposed scenario (20% reduction in current stage IV)	
	Number of p.t	Number of p.t	Increase in %	Number of p.t	Increase in %	Number of p.t	Increase in %
*2023*	140	290	108%	251	80%	193	38%
*2024*	145	301		261		201	
*2025*	148	308		267		205	
*2026*	152	315		273		210	
*2027*	155	322		279		215	

p.t.: patients

The survival outcomes at year five varies by the diagnostic stages. In the current scenario, with approximately 991 to 1100 new cases diagnosed annually from 2023 to 2027, there would be 140 to 155 patients expected to be alive at 5 years post diagnosis. In the proposed scenario, with the same number of new cases each year, there would be 290 to 322 patients anticipated to survive up to year 5 post diagnosis due to the stage shift (70% of the shift in stage IV cancer) upon diagnosis. In the proposed scenario, the 5-year survival rate could be improved to 29% compared to 14% in the current scenario. This translated into a total additional 795 out of 5246 patients with pancreatic cancer surviving up to year 5 (Table 3).

**Table 4:** Results of survival outcomes by remoteness levels

		Metropolitan	Regional centres	Large rural towns	Medium/small rural towns^*^
2023	Current	113	8	8	10
	Proposed	235	17	17	21
2024	Current	118	8	9	10
	Proposed	244	17	18	22
2025	Current	121	8	9	10
	Proposed	250	18	18	22
2026	Current	124	9	9	11
	Proposed	257	18	18	22
2027	Current	127	9	9	11
	Proposed	263	18	18	23

Propose scenario refers to the base case scenario where 70% of stage IV cases could be shifted to Stage I-II with a screening tool

MM1: Metropolitan; MM2: Regional centres; MM3: large rural towns; MM4: medium rural towns; MM5: small rural towns; MM6: remote communities; MM7: very remote communities [35]. ^*^MM4 and over are combined

In the proposed scenario with a targeted surveillance program in Victoria, more people with pancreatic cancer would survive up to 5 years, irrespective of different remoteness levels. However, the 5-year survival outcomes, as depicted in Table 4, varied across different remoteness levels according to the current and proposed scenarios. In the proposed scenario, it is anticipated that individuals residing in metropolitan would have a greater raw number of cancer survivors at the 5-year mark due to the higher population density.

### Gaps in workforce supply

We identified significant gaps in the key workforce supply involved in pancreatic cancer treatment and management (Table 5). For example, large rural towns (MM3), and medium/small rural towns and beyond (MM4 and over) in Victoria appeared to have significantly reduced number of workforce involved in pancreatic cancer treatment and management, suggesting in these areas (i) patients have to travel to at least the regional centres or large rural towns (MM 3/2) to receive their pancreatic cancer care; or (ii) health professionals are servicing in a part-time capacity.

**Table 5:** Descriptive statistics of the key health workforce and estimated new cases by remoteness levels

Current scenario							
	Endocrinology	Gastroenterology and Hepatology	Palliative medicine	Pain medicine	General surgery^^^	Radiation oncology	Medical oncology
Metropolitan	1635	1992	463	236	3174	734	1588
Regional centres	55	49	53	30	274	87	166
Large rural towns	7	19	11	0	296	28	96
Medium/small rural towns^*^	7	15	3	0	176	0	3
**Estimated increase in Stages I-II cases by remoteness levels (base case)**							
	**2023**	**2024**	**2025**	**2026**	**2027**		
Metropolitan	324	337	346	354	363		
Regional centres	23	24	24	25	25		
Large rural towns	24	24	25	25	25		
Medium/small rural towns^*^	29	29	31	31	31		

MM1: Metropolitan; MM2: Regional centres; MM3: large rural towns; MM4: medium rural towns; MM5: small rural towns; MM6: remote communities; MM7: very remote communities [35]. ^*^MM4 and over are combined. ^^^Note that only a proportion of general surgeons specialise in pancreatic cancer procedures, and the specialty of the general surgeons may be associated with the differences in survival outcomes of patients undergoing the surgery

The distribution of radiation oncologists and physicians (palliative medicine, medical oncologist, and endocrinology) exhibited a significant imbalance in remoteness levels, according to their primary registration location.

With the workforce supply projection, it is expected that health professionals from regional centres or large rural towns (MM 2/3) may have more impact on their workload due to (i) the predicted increase in pancreatic cancer Stage I-II cases in rural/remote areas (MM 3–7); and (ii) insufficient workforce supply in rural/remote areas (MM 3–7) (Table 5 and Supplementary document).

Additional analyses by Local Government Areas are provided in the Supplementary document.

## Discussion

According to the federal government’s labour forecasts, workforce demand in the health sector will increase by 14.9 per cent over the next five years [44]. However, it is also forecasted that the healthcare industry will experience major workforce shortages over the coming years due to an ageing workforce and current retention challenges, especially in regional areas [44]. As emphasised by the World Health Organization (WHO), the lack of human resources for health to meet the present and rising population demands globally is a significant hurdle to attaining the Sustainable Development Goals. Health systems continue to face a wide range of complicated and varied difficulties with regard to human resources for health, notwithstanding modest success in improving the total health workforce aggregates globally. These limitations include a lack of qualified workforce in terms of numbers as well as differences in the skill mix, unequal geographic distribution, problems with inter-professional collaboration, ineffective resource use, and fatigue [45–52]. Hence, effective management of the workforce is of paramount importance to satisfy the needs of human resources within health systems and to enhance capabilities at regional and global levels.

Early diagnosis is crucial in pancreatic cancer due to its rapid progression from stages I to IV [38]. Risk-based screening for pre-cancerous individuals has the potential to improve the poor prognosis and extend survival rates of this disease. Our study findings highlighted a potential significant survival gain from screening and early detection of pancreatic cancer. Our modelling results indicated that early diagnosis of pancreatic cancer is expected to bring significant survival benefits by diagnosing an additional 400 and 445 patients in the earlier stages from 2023 to 2027). Our study showed that assuming 70% of the shift from stage IV to stages I-II would result in additional 795 patients who survive up to year 5 out of 5246 new cases expected in the next five years, representing up to 108% increased survival from the current scenario (e.g. 140 versus 290 patients surviving to year five post diagnosis). Achieving the expected survival benefits is subject to timely and sufficient access to quality healthcare. The low 5-year survival in pancreatic cancer together with the survival gain in anticipation from early detection, warrant investment in screening of high-risk populations to significantly advance the current care paradigm.

The availability, accessibility, acceptability, and quality of health workers play a crucial role in providing quality health services [53]. However, just having health workers available is not enough. Ensuring an equitable distribution and accessibility of health workers, along with their required competency, motivation, and empowerment to deliver quality care that meets the sociocultural expectations of the population, are also critical factors. Based on our workforce modelling, for example, while the overall number of additional stage I-II cases diagnosed in 2023 may be manageable by the current workforce, the distribution of the existing workforce raises concerns about equitable health (i.e. health professionals in the outer regional areas and beyond may experience a more pronounced workload increase compared to their metropolitan counterparts). The value of introducing initiatives for early diagnosis of pancreatic cancer may vary for people depending on their residing location and accessibility to medical professionals. While some medical specialities may practice at multiple locations, limitations in the available workforce data means there is no data regarding the location of their non-primary workplace, not the time fractions spent at these locations. Despite some progress in improving health workforce availability, there is still a need to mobilise resources for the workforce agenda as part of broader efforts to strengthen and adequately finance health systems in certain areas of Victoria and throughout Australia.

To establish a robust and efficient health workforce, ensuring that health workers’ supply and skills match the population’s present and future needs is crucial. This is especially important given the growing burden of noncommunicable diseases and chronic conditions on health systems worldwide [54], which also requires a shift towards patient-centred care, community-based health services, and personalised long-term care [55]. Achieving the necessary quality, quantity, and relevance of the health workforce calls for policy and funding decisions that align with these evolving needs. Past initiatives in health workforce development have yielded positive results, with countries that have addressed their health workforce challenges observing improvements in health outcomes, supported by compelling evidence [33, 56].

The multidisciplinary team, typically consisting of health professionals involved in diagnosis, treatment, and supportive care (including palliative and pain specialist care) is recommended for pancreatic cancer care to ensure patients receive optimal treatment and to mitigate variations in treatment [57]. Previous studies have shown that presentation at a multidisciplinary team meeting can change the proposed treatment strategy for up to 25% of pancreatic cancer patients and was associated with increased survival and decreased socioeconomic disparity in treatment [58–60]. The lack of key health professionals in at a local level can have significant health consequences. Greater use of telemedicine and teleconferences may help to address gaps relating to some medical professions and ensure all the medical expertise is present at multidisciplinary meetings.

A study conducted in Australia simulated the use of the Web-based QCancer 10-year risk algorithm for various types of cancer including pancreatic cancer [61]. The findings of that study suggest that the algorithm may be a useful tool for patients with complex medical histories [61]. Whilst a notable lack of a recommended screening tool for pancreatic cancer exists in Australia, prompting research efforts to bridge this gap in pancreatic cancer care and enable early detection and diagnosis. Considering the abundance of risk prediction tools available for pancreatic cancer [23], there is a promising prospect of modifying and externally validating these tools for broader use in local contexts.

We used publicly available disease incidence and workforce data to model the potential demand and supply in pancreatic cancer treatment and management in a granular way for the next five years by considering the trend in changes. The following limitations should be considered when interpreting the findings. First, we did not examine the age distribution of the current workforce. Therefore, it is unknown whether the health workforce experienced aging, too, as the general population, which may impact the future workforce supply. However, the trend in the workforce changes over the past may have accounted for it to some extent. Second, we did not examine the diagnostic yields of screening tools in identifying high-risk people with potential pancreatic cancer, rather this simulation study assesses the health workforce readiness (e.g. the most optimistic and pessimistic scenarios) in response to a targeted surveillance program. Third, workforces that may participate in pancreatic cancer diagnosis, treatment and management are not exhaustively modelled in the current study; for example, radiologists, psychologists, nurses, and allied health practitioners (dietitians, diabetes educators) were excluded from the analysis. Due to the lack of evidence for the proportion of patients requiring each type of treatment and the clinical heterogeneity in potential treatment pathways (e.g., patients undergoing surgery, neoadjuvant therapy, chemoradiation therapy, etc.), simulating patients receiving individual types of treatment would require substantial assumptions subject to considerable uncertainties. As a result, we have simulated the number of new cases by stage, and any subsequent workforce impact stemming from a specific type of treatment (including recurrence) will be based on these case numbers. Fourth, the presented study did not examine the impact of diagnostic logistics due to the stage shift towards early stage of pancreatic cancer. However, the stage shift is unlikely to exert significant impact on the health workforce involved in the diagnostic phase of the condition, due to the fact that we only assumed stage shift occurred in the incident pancreatic cancer case within that year (regardless these cases will be diagnosed in that year, only with a difference in diagnostic stage).

Due to the utilisation of the US survival rate for pancreatic cancer, the model slightly overestimated the 5-year survival in the current scenario (14% vs. 12.2%) [23]. However, the aggregated 5-year survival across all stages at diagnosis were fairly comparable in US and Australia (11% VS. 12.2%) [23, 62]. However, the aggregated 5-year survival across all stages at diagnosis was fairly comparable in the US and Australia (11% vs. 12.2%). There are no significant differences in the 5-year survival rate in the global context for pancreatic cancer [29]. Finally, due to the limitation of health workforce data, we were unable to ascertain how areas by remoteness levels without targeted workforce are serviced at present to quantify the gaps in workforce supply more accurately. Additionally, we cannot determine the extent to which the hub and spoke model of care and telehealth have been adopted in the process of care for people with pancreatic cancer. Nevertheless, we believe our estimation can serve as the worst-case scenario for healthcare workforce requirements.

## Conclusions

Improving the early detection and diagnosis of pancreatic cancer is expected to bring significant survival and morbidity benefits although there are workforce distribution imbalances in Victoria that may affect the ability to achieve the anticipated survival gain. Dedicated approaches are required to ensure access to a multidisciplinary healthcare workforce delivering screening and treatment in some regions of Victoria. Investing in screening and decision-support tools for high-risk populations could facilitate pancreatic cancer’s early detection and diagnosis.

### Electronic supplementary material

Below is the link to the electronic supplementary material.

Supplementary Material 1

## Data Availability

All data analysed during this study are publicly available from corresponding references cited in this published article [and its supplementary information files].

## Abbreviations

NSW: New South Wales
MMM: Modified Monash Model
ABS: Australian Bureau of Statistics
LGA: Local Government Areas
