# Supplementary material for: Simulating the healthcare workforce impact and capacity for pancreatic cancer care in Victoria: a model-based analysis
Source: BMC Health Serv Res. 2024 Feb 23;24:239. doi: 10.1186/s12913-024-10722-9 (PMC10893744; doi:10.1186/s12913-024-10722-9)
Supplement: Supplementary file 1 — Supplementary Material 1 [file 12913_2024_10722_MOESM1_ESM.docx]

# Supplementary Tables

## Supplementary Table 1. Incidence of pancreatic cancer

| **Year** | **Incidence(age-standardised)** |
| --- | --- |
| 2021 | 14.5 |
| 2020 | 14.8 |
| 2019 | 14.4 |
| 2018 | 15.0 |
| 2017 | 15.6 |
| 2016 | 15.4 |
| 2015 | 15.4 |
| 2014 | 14.8 |
| 2013 | 13.3 |
| 2012 | 13.7 |
| 2011 | 13.7 |
| 2010 | 14.4 |
| 2009 | 13.4 |
| 2008 | 12.9 |
| 2007 | 14.4 |
| 2006 | 12.9 |
| 2005 | 12.2 |
| 2004 | 12.9 |
| 2003 | 11.8 |
| 2002 | 11.7 |
| 2001 | 13.1 |
| 2000 | 11.9 |

Footnote: there is no incidence data by stage for pancreatic cancer in Australia

## Supplementary Table 2. Pancreatic cancer diagnostic stages examined

|  | **Current scenario^*^** | **Proposed scenario** | | |
| --- | --- | --- | --- | --- |
|  |  | 20% shift | 50% shift | 70% shift |
| Stages I-II | 20% | 35% | 50% | 60% |
| Stage III | 30% | 25% | 25% | 25% |
| Stage IV | 50% | 40% | 25% | 15% |

**^*^**Reference: Cancer Council NSW <https://www.cancercouncil.com.au/pancreatic-cancer/diagnosis/staging-prognosis/> Accessed on 15 March 2023.

For example, in the current scenario, 50% of cases were diagnosed in Stage IV, with 20% of shift towards earlier stages, the Stages IV could reduce to 40% (from 50%) while Stages I-II could increase to 35% assuming Stage III remain unchanged

## Supplementary Table 3. Pancreatic cancer 5-year survival rate

| **Stage** | **5-year Relative Survival Rate** |
| --- | --- |
| Localised (stages I--II) | 42% |
| Regional (stage III) | 14% |
| Distant (stage IV) | 3% |
| All stages combined | 11% |

Reference: American Cancer Society, <https://www.cancer.org/cancer/types/pancreatic-cancer/detection-diagnosis-staging/survival-rates.html>. Accessed on 15 March 2023.

The 5-year survival by diagnostic stage was not available in Australia.

## Supplementary Table 4. Increase in Stages I-II cases by remoteness levels (scenario analysis)

| **50% of stage shift** | | | | | |
| --- | --- | --- | --- | --- | --- |
|  | **2023** | **2024** | **2025** | **2026** | **2027** |
| Metropolitan | 244 | 254 | 260 | 267 | 273 |
| Regional centres | 17 | 18 | 18 | 19 | 19 |
| Large rural towns | 18 | 18 | 19 | 19 | 19 |
| Medium/small rural towns | 22 | 22 | 23 | 23 | 23 |
| **20% of stage shift** | | | | | |
|  | **2023** | **2024** | **2025** | **2026** | **2027** |
| Metropolitan | 124 | 129 | 132 | 135 | 139 |
| Regional centres | 9 | 9 | 9 | 9 | 10 |
| Large rural towns | 9 | 9 | 9 | 9 | 10 |
| Medium/small rural towns | 11 | 12 | 12 | 12 | 12 |

MM1: Metropolitan; MM2: Regional centres; MM3: large rural towns; MM4: medium rural towns; MM5: small rural towns; MM6: remote communities; MM7: very remote communities (35). *MM4 and over are combined

**Supplementary Table 5. Workforce Supply data for the key health professionals involved in pancreatic cancer treatment**

| Year | **General surgery** | | | **Radiation oncology** | | | **Medical oncology** | | |
| --- | --- | --- | --- | --- | --- | --- | --- | --- | --- |
|  | **100%*** | **50%^** | **20%^** | **100%** | **50%** | **20%** | **100%** | **50%** | **20%** |
| *2023* | 4135 | 2068 | 827 | 914 | 457 | 183 | 2104 | 1052 | 421 |
| *2024* | 4248 | 2124 | 850 | 946 | 473 | 189 | 2241 | 1121 | 448 |
| *2025* | 4364 | 2182 | 873 | 979 | 490 | 196 | 2388 | 1194 | 478 |
| *2026* | 4483 | 2242 | 897 | 1013 | 507 | 203 | 2544 | 1272 | 509 |
| *2027* | 4605 | 2303 | 921 | 1048 | 524 | 210 | 2711 | 1356 | 542 |

*The number of health professionals according to the primary specialty. ^Assuming 50% and 20% of the respective professionals with speciality in pancreatic cancer.

## Supplementary Table 6. Areas with no workforce involved in pancreatic treatment and management

| **Local government areas (n=25)** | **Physician-endocrinology** | **Physician-gastroenterology and hepatology** | **Palliative medicine** | **Pain medicine** | **Surgery-general surgery** | **Radiation oncology** | **Physician-medical oncology** |
| --- | --- | --- | --- | --- | --- | --- | --- |
| Cardinia | 0 | 0 | 0 | 0 | 0 | 0 | 0 |
| Nillumbik | 0 | 0 | 0 | 0 | 0 | 0 | 0 |
| Surf Coast | 0 | 0 | 0 | 0 | 0 | 0 | 0 |
| Golden Plains | 0 | 0 | 0 | 0 | 0 | 0 | 0 |
| Hepburn | 0 | 0 | 0 | 0 | 0 | 0 | 0 |
| Indigo | 0 | 0 | 0 | 0 | 0 | 0 | 0 |
| Loddon | 0 | 0 | 0 | 0 | 0 | 0 | 0 |
| Mount Alexander | 0 | 0 | 0 | 0 | 0 | 0 | 0 |
| Pyrenees | 0 | 0 | 0 | 0 | 0 | 0 | 0 |
| Towong | 0 | 0 | 0 | 0 | 0 | 0 | 0 |
| Macedon Ranges | 0 | 0 | 0 | 0 | 0 | 0 | 0 |
| Moyne | 0 | 0 | 0 | 0 | 0 | 0 | 0 |
| Benalla | 0 | 0 | 0 | 0 | 0 | 0 | 0 |
| Central Goldfields | 0 | 0 | 0 | 0 | 0 | 0 | 0 |
| Moira | 0 | 0 | 0 | 0 | 0 | 0 | 0 |
| Alpine | 0 | 0 | 0 | 0 | 0 | 0 | 0 |
| Buloke | 0 | 0 | 0 | 0 | 0 | 0 | 0 |
| Corangamite | 0 | 0 | 0 | 0 | 0 | 0 | 0 |
| Gannawarra | 0 | 0 | 0 | 0 | 0 | 0 | 0 |
| Hindmarsh | 0 | 0 | 0 | 0 | 0 | 0 | 0 |
| Mansfield | 0 | 0 | 0 | 0 | 0 | 0 | 0 |
| Strathbogie | 0 | 0 | 0 | 0 | 0 | 0 | 0 |
| Unincorporated Vic | 0 | 0 | 0 | 0 | 0 | 0 | 0 |
| West Wimmera | 0 | 0 | 0 | 0 | 0 | 0 | 0 |
| Yarriambiack | 0 | 0 | 0 | 0 | 0 | 0 | 0 |

# **Supplementary Figures**

## Supplementary Figure 1. Predicted pancreatic cancer cases in Victoria in 2023


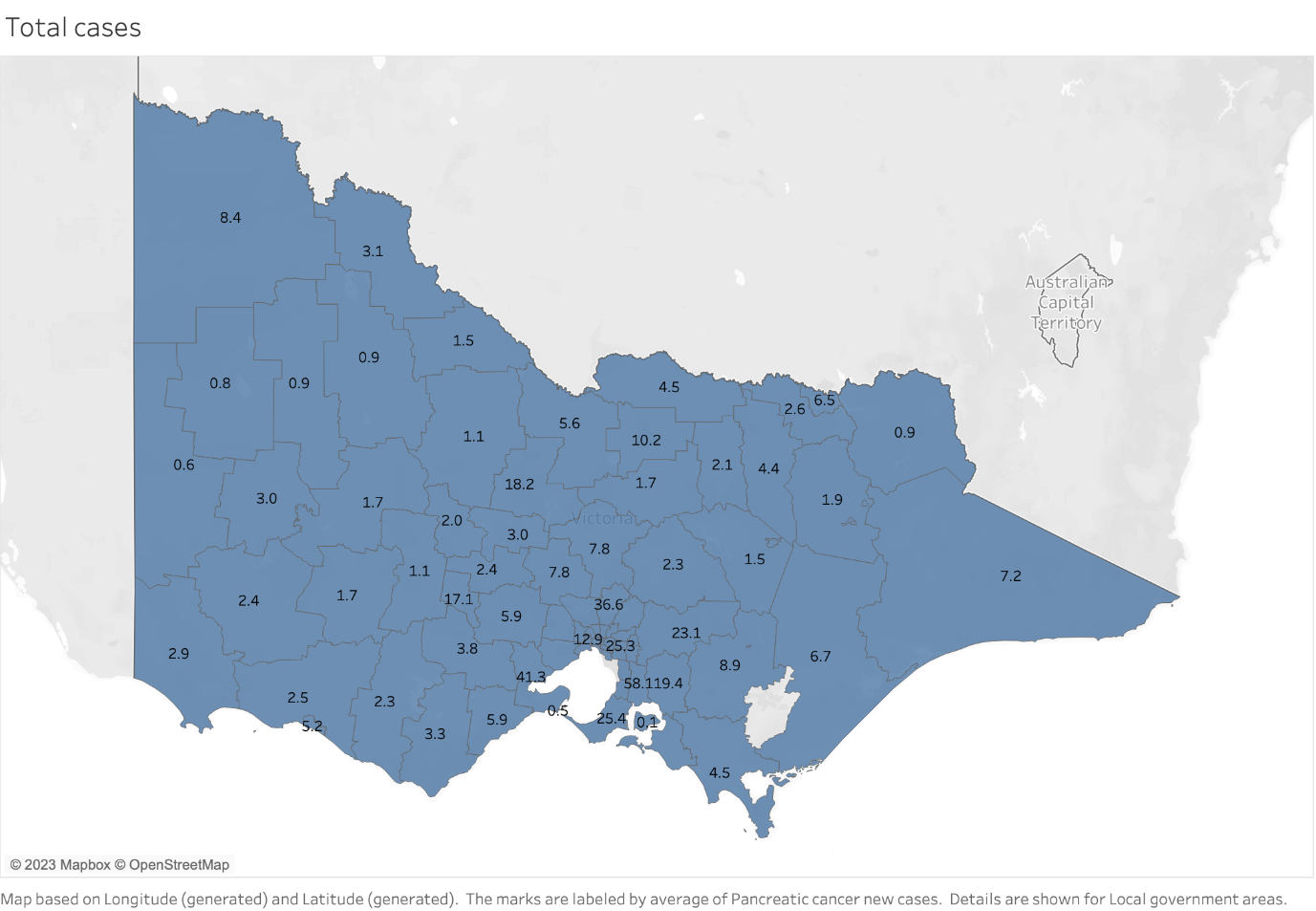


## Supplementary Figure 2. Predicted increased pancreatic cases in 2023 (70% of the shift towards earlier stages)


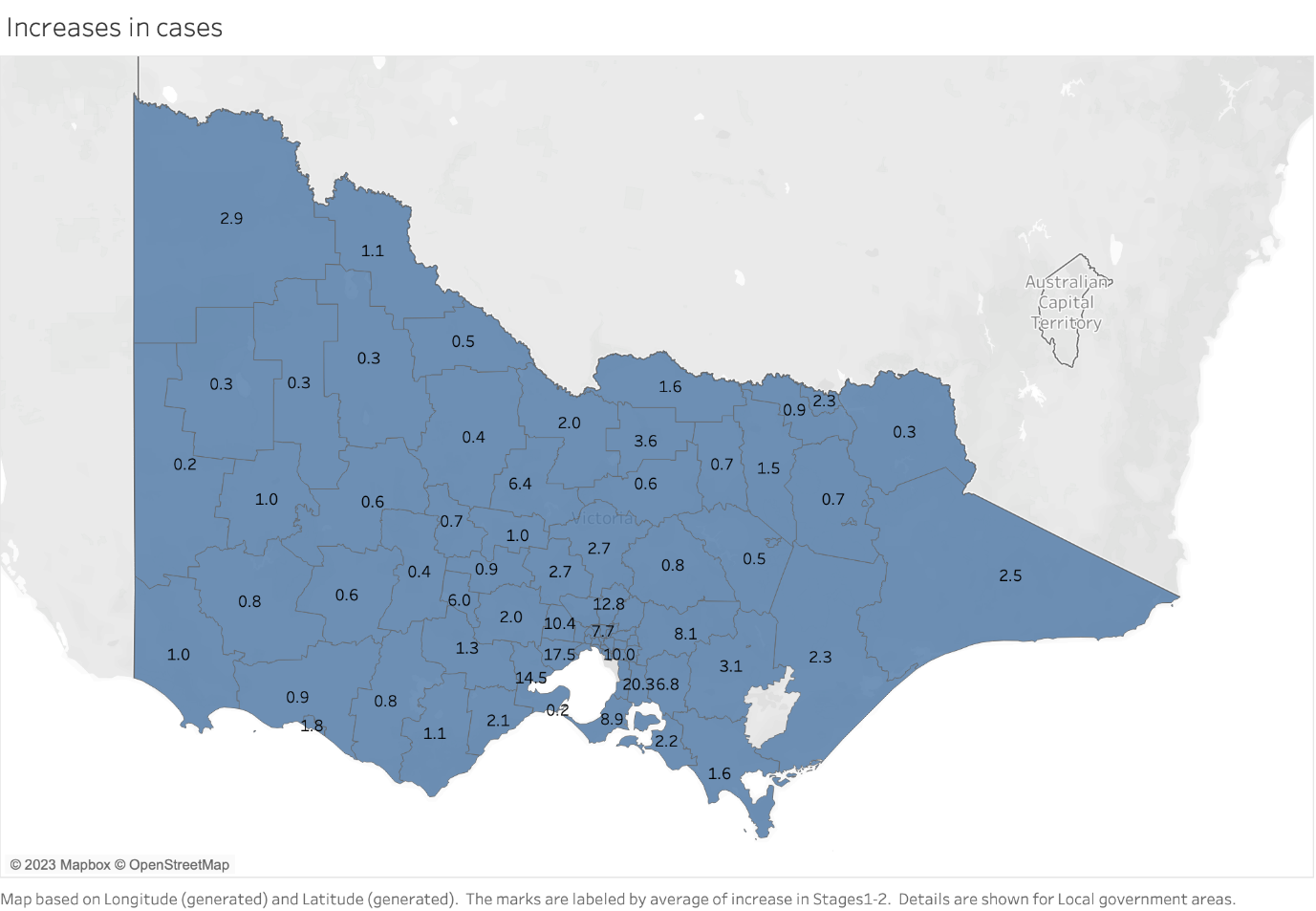


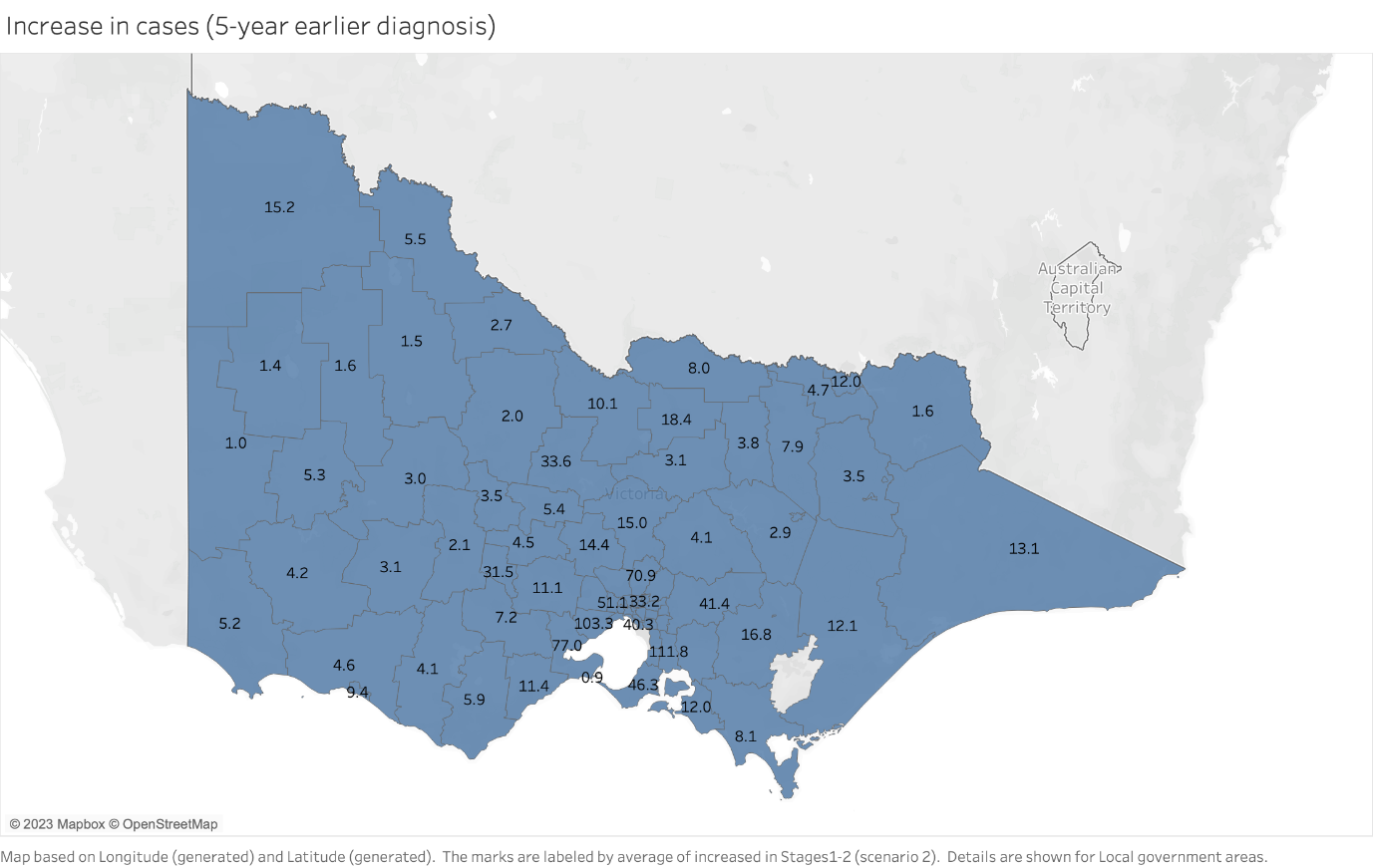


The upper figure shows the number of additional early stages cases if assuming the stage shift within a year; the lower figure illustrates the additional case number assuming stage shift by 5-year.

Supplementary Figure 3. Distribution of radiation oncologists in Victoria
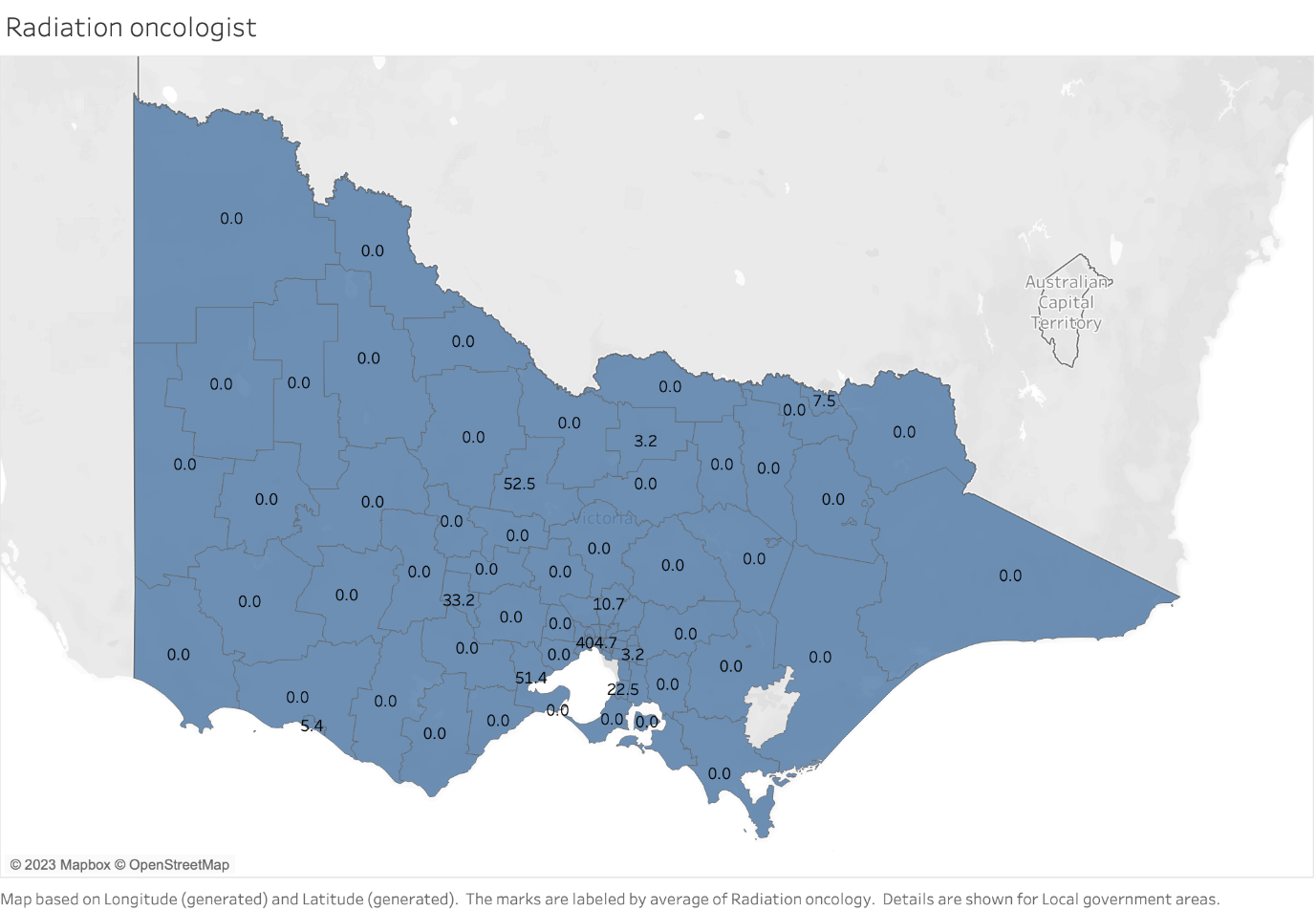


The distribution represents their primary registration location, where they could have multiple locations of practice.

Supplementary Figure 4. Distribution of palliative medicine physicians in Victoria
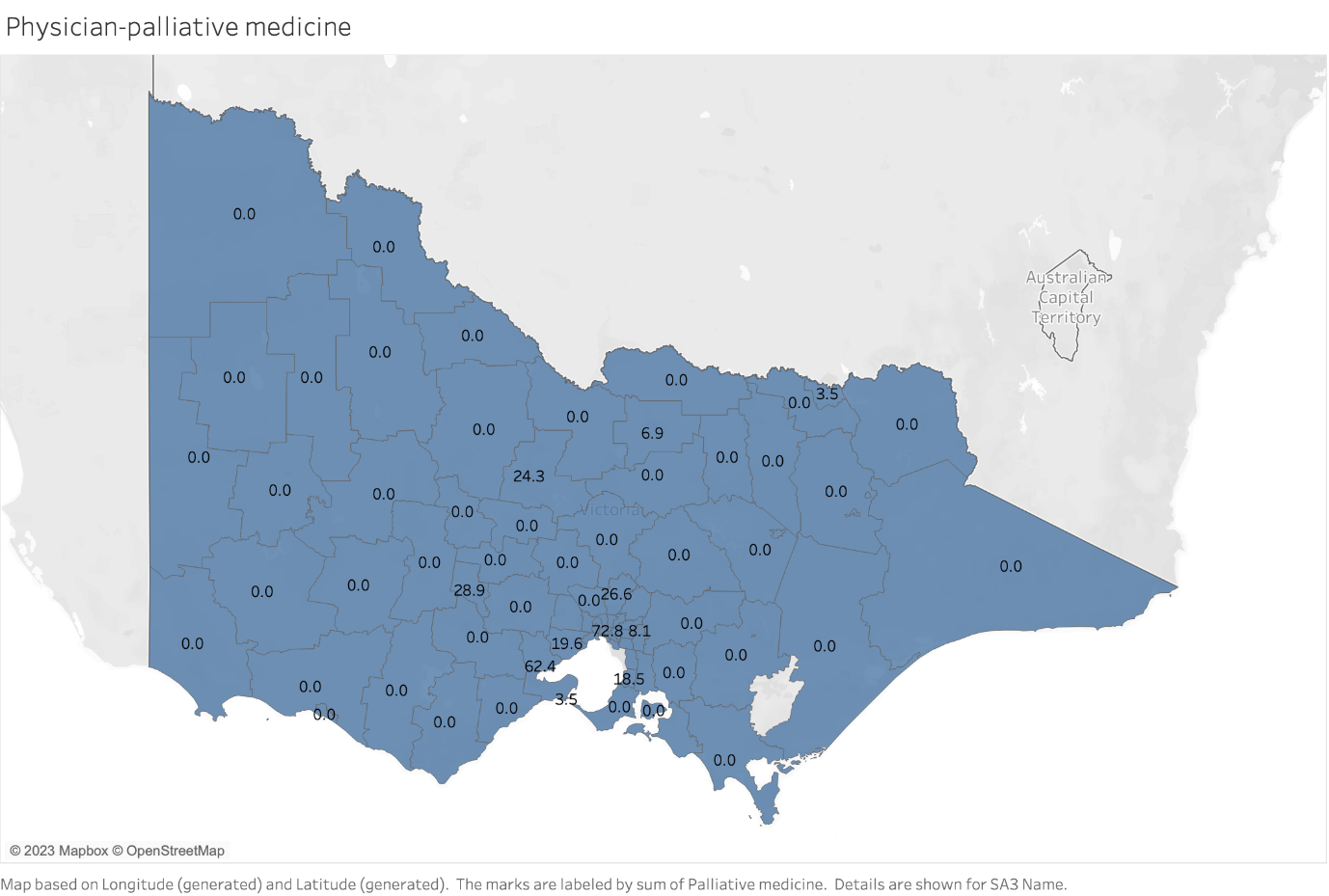


The distribution represents their primary registration location where they could have multiple locations of practice.
